# ScRNA-seq combined with ATAC-seq analysis to explore the metabolic balance mechanism of CCl4-induced liver inflammatory injury

**DOI:** 10.3389/fimmu.2025.1600685

**Published:** 2025-06-16

**Authors:** Hui Liu, Yisha Zhang, Shoubin Ning

**Affiliations:** ^1^ Department of Gastroenterology, Air Force Medical Center, Air Force Medical University, Beijing, China; ^2^ Department of Internal Medicine, First Rongjun Youfu Hospital of Shandong, Jinan, China

**Keywords:** CCl4, chronic liver injury, histone acetylation, fatty acid metabolism, ZHX2, ZBTB20

## Abstract

**Background:**

Drug-induced liver injury (DILI) can provoke inflammation and fibrosis in the liver, potentially leading to severe liver diseases and mortality; however, effective treatments for liver fibrosis remain elusive. The objective of this study was to explore the cellular metabolic mechanism after carbon tetrachloride (CCl4)-induced liver injury.

**Methods:**

Initially, we conducted a comprehensive analysis of ATAC-seq, RNA-seq, and scRNA-seq datasets derived from CCl4-induced chronic liver injury in mice. Subsequently, functional enrichment analysis and transcription factor analysis were performed. Finally, the expression changes of key substances and transcription factors were verified by cell and animal experiments.

**Results:**

Our investigation uncovered that hepatocyte histone acetylation intensified with prolonged injury durations. Subsequent functional enrichment analysis identified that fatty acid metabolism as the predominant pathway implicated in hepatocyte damage. The tricarboxylic acid cycle in hepatocytes exhibited partial slowdown and the mitochondrial electron transport chain (ETC) was inhibited in the early stage of CCl4-induced chronic injury. However, in the later stage of injury, there was a gradual restoration of the ETC functionality, coupled with an enhanced capacity for *de novo* synthesis of fatty acids. This process of metabolic equilibrium restoration may be related to acute lipid accumulation during liver injury repair. Transcription factor analysis found that Zhx2, a crucial suppressor of ETC, experienced sustained increases in chromatin accessibility within injured hepatocytes, but its expression level increased first and then decreased. The key transcriptional repressor Zbtb20 could inhibit the expression of Zhx2, and its expression trend corresponded to that of Zhx2. Cellular experiments demonstrated that CCl4 induced upregulation of acetyl-CoA, Zhx2 and Zbtb20 in a time-dependent manner. The levels of acetyl-CoA and Zbtb20 increased with the duration of injury in animal experiments, but Zhx2 showed a rise in expression only at week 3, while expression returned to normal levels after week 6.

**Conclusion:**

Our findings contribute to the understanding of the evolution and underlying CCl4-induced inflammatory mechanisms governing hepatocyte inflammatory injury and the subsequent metabolic shift from imbalance toward balance under chronic CCl4 exposure, offering novel perspectives and directions for targeted therapeutic interventions in DILI.

## 1 Introduction

The liver is a vital organ with intricate physiological functions. It plays a pivotal role in systemic metabolism through processes such as nutrient absorption, detoxification, and excretion. Hepatocytes, the most abundant cell type within the liver, constitute approximately 60% of the total cellular population. These hepatocytes are crucial for synthesizing plasma proteins, detoxifying exogenous substances, and regulating the urea cycle (1). In healthy liver, hepatocytes are radially arranged around the central vein, separated by sinusoids containing hepatic stellate cells (HSCs), Kupffer cells, and extracellular components. When liver cell damage is induced by certain stimuli, a fibrotic response is activated, leading to the synthesis of type I collagen by HSCs. Repeated injury and activation of fibrogenesis result in chronic fibrogenic activity, with type I collagen accumulating in the extracellular matrix (ECM) surrounding the lobules. This accumulation leads to liver fibrosis and impairs liver function (2).

Drugs can induce liver inflammation and fibrosis through drug-induced liver injury (DILI) (3), which can progress to cirrhosis, hepatocellular carcinoma, and even death if not treated in time (4). Currently, there is no definitive treatment for liver fibrosis other than liver transplantation, which poses a significant threat to the health of patients (5). Therefore, exploring the physiological and pathological mechanisms, as well as key cellular processes and genes involved in liver responses to DILI, is crucial for improving treatment strategies for chronic liver damage. The two most common models used in DILI research are acetaminophen (APAP) and carbon tetrachloride (CCl4). Unlike APAP, which is often used to induce acute liver injury, CCl4 can be utilized to simulate chronic liver injury diseases. Previous studies have demonstrated that low doses of CCl4 administered twice weekly for 2–4 weeks can cause persistent liver damage (6). The hepatotoxic mechanism of CCl4 involves three critical phases mediated by CYP450. Initially, CCl4 undergoes metabolic activation in hepatocytes to generate the highly reactive CCl₃•. This radical directly attacks cellular macromolecules, disrupting protein functions, and impairing lipid metabolism to promote fatty degeneration. Subsequent oxygenation of CCl₃• produces CCl₃OO•, which initiate lipid peroxidation cascades that degrade polyunsaturated fatty acids and compromise membrane integrity across cellular compartments. The resultant membrane permeability alterations drive mitochondrial dysfunction, endoplasmic reticulum stress, and ionic imbalance, thereby mediating hepatotoxicity (7–10). However, the metabolic mechanism underlying CCl4-induced chronic liver injury remains not fully understood.

In this study, we first collected datasets related to CCl4-induced chronic liver injury using Assay for Transposase-Accessible Chromatin sequencing (ATAC-seq), RNA sequencing (RNA-seq), and single-cell RNA sequencing (scRNA-seq). We analyzed the chromatin accessibility of hepatocytes and explored the activity of histone-related pathways in these cells. Gene Set Enrichment Analysis (GSEA) and Gene Ontology (GO) enrichment analysis confirmed changes in enriched pathways in hepatocytes during CCl4-induced chronic liver injury over time. Additionally, we investigated the metabolic balance mechanisms and key metabolites involved in CCl4-induced chronic liver injury. Finally, through cell and animal experiments, we verified the concentration changes of acetyl coenzyme A (acetyl-CoA), Zbtb20, and Zhx2 with the extension of CCl4-treated time. These findings provide a direction for further exploring the physiological and pathological processes and mechanisms of DILI and lay a theoretical foundation for preclinical research on targeted therapy.

## 2 Materials and methods

### 2.1 Data collection and related information

The data of scRNA-seq was collected from the study by Guo et al. (11). Mice were injected with 0.5 mL/kg CCl4 twice a week (CCl4: oil = 1: 4), and data for 3, 6, and 10 weeks were employed. The available fastq data of the ATAC-seq dataset GSE233080 was downloaded from the European Nucleotide Archive (ENA) database. RNA-seq datasets GSE222576 and GSE207855 for chronic stimulation of CCl4; GSE144600 for acute CCl4 stimulation; thioacetamide (TAA), choline-deficient diet with 0.5% DL-ethionine supplement (CDE) damage scRNA-seq dataset GSE200366 (12); and 2,3,7,8-tetrachlorodibenzo-p-dioxin (TCDD) damage scRNA-seq dataset GSE148339 were obtained from the Gene Expression Omnibus (GEO) database (13). GSE233080 contains 0-day control and CCl4-injured mice (injected with 2.5 mL/kg of 10% CCl4 twice a week) (14). GSE222576 includes controls and mice injected with CCl4 for 2, 4, 6, 8, and 10 weeks, with an injection condition of 1.6 g/kg twice a week (CCl4: oil = 1: 3) (15). Mice in GSE207855 were injected with 0.5 mL/kg three times a week for 6 weeks (CCl4: oil=1:6) (16). The data of CCl4-treated and control mice in GSE144600 was used, and mice were injected with 5 mL/kg of 10% CCl4 (17). Notably, variations in CCl4 administration protocols exist across datasets. To address this, we standardized units during data processing. Importantly, the cumulative CCl4 dosage correlates with injury severity, whereas differences in dosing schedules or dilution ratios did not confound our assessment of liver injury progression in this study.

### 2.2 ATAC-seq analysis

Fastp (v0.23.4) was used to process ATAC-seq data, which was then compared and deduplicated through BWA (v0.7.18) and SamBamba (v1.0.1), then sorted by Samtools (v1.20). MACs2 (v2.2.9.1) software was applied for peak calling, and finally, the DiffBind (v3.12.0) package, with the edgeR method and default parameters, was utilized for differential analysis to obtain the peaks of difference between different groups. The above analysis used the default parameters. Visualization was conducted with the software DeepTools (v3.5.5).

### 2.3 ScRNA-seq analysis

We performed quality control (QC) on scRNA-seq data using the Seurat (v5.0.1) package. The screening criteria were nFeature_ RNA > 508, percent.mt < 10, nCount_RNA < 7000, nFeature_RNA < 4000, and nCount_RNA > 2000. The threshold values for these screening criteria were derived from the original literature (11). The decontX (v1.0.0) package was used to remove free RNA, and the NormalizeData function was utilized for standardization. The number of hypervariable genes was set to 3000. In ScaleData, mitochondrial proportion and nCount_RNA was regressed out. The harmony (v1.2.0) package was employed to remove batch effects between samples. 20 principal components (PCs) were used for dimensionality reduction clustering, with a resolution of 0.3. The cells were annotated using markers from the original research in the dataset (11). Seurat and ggplot2 (v3.4.4) packages were applied for visualization.

### 2.4 GSEA and enrichment analysis of differentially expressed genes

DEGs in RNA-seq data were identified using the limma (v3.58.1) package (18), while DEGs in scRNA-seq data were analyzed by FindMarkers function of the Seurat package. GSEA was performed through the clusterProfiler (v4.10.0) package (19). The clusterProfiler package was also utilized to analyze DEGs in both RNA-seq and scRNA-seq data, and pathways with significant normalized enrichment score (NES) in RNA-seq and scRNA-seq data were screened (p < 0.05). Visualization was conducted using the enrichplot (v1.22.0) package.

### 2.5 Expression of key genes in different energy metabolism pathways

The related genes involved in fatty acid (FA) and glucose metabolism were identified by consulting existing literature, and the differential expression of these genes was analyzed among different groups of hepatocytes using scRNA-seq data. The analysis was conducted using the Seurat package, and visualization was performed through the ggplot2 package.

### 2.6 Expression analysis of *Zhx2* and transcription factors analysis

Previous studies have demonstrated that the transcription factor *Zhx2* effects acute liver injury through two primary mechanisms: First, *Zhx2* binds to the conserved motif in the 5’-UTR of ETC genes and occupies the ETC gene promoter, directly suppressing ETC gene transcriptional activity; Second, *Zhx2* enhances the transcriptional activation of FBXW7, which in turn reduces the protein level of PGC-1α and consequently limits mitochondrial biogenesis (20). Building on these findings, our study further explored the role of *Zhx2* in the setting of chronic liver injury, revealing novel mechanistic insights into the mechanisms by which *Zhx2* dynamically regulates both metabolic adaptation and fibrotic remodeling during sustained hepatotoxic stress. We analyzed the expression changes of *Zhx2* and ETC-related genes in scRNA-seq data. Additionally, we explored and compared the expression changes of *Zhx2* and ETC-related genes in scRNA-seq datasets of chronic liver injury induced by other drugs, with our previous analysis.

We identified TFs that may regulate *Zhx2* by intersecting ATAC-seq data, DEGs and TFs obtained from the AnimalTFDB database. These TFs were searched in the Cistrome database to examine if they have a peak at *Zhx2* locus. The results were visualized using deeptools software.

### 2.7 Functional changes of chronic liver injury in different periods

ScRNA-seq hepatocyte data were analyzed using the ReactomeGSA (v.1.16.1) package to investigate comparative changes in function over different periods, and the results were visualized as heat maps.

### 2.8 Cell culture and treatment

The AML-12 mouse hepatocyte cell line was obtained from the American Type Culture Collection (CRL-2254, ATCC, USA) and were cultured in DMEM/F-12 medium (1:1) (HyClone, Thermo Scientific, MA, USA) supplemented with 10% fetal bovine serum (HyClone, Thermo Scientific, MA, USA), 1% ITS Media Supplement (Cat. No. C0341, Beyotime, China) and 40 ng/ml dexamethasone (Cat. No. ST1254, Beyotime, China). Cells were grown in a 37 °C humidified atmosphere containing 5% CO2. AML-12 cells were treated with 10 mM CCl4 (Cat. No. 56-23-5, Macklin, China) or solvent for 24 h, 48 h and 72 h, respectively (16, 17).

### 2.9 Enzyme-linked immunosorbent assay

AML-12 cells or liver tissue were collected and put into pre-cooled normal saline followed by ultrasonic trituration and centrifugation at 3000 rpm for 10 min to obtain supernatant. Acetyl-CoA in the supernatant was measured by ELISA Kits (Cat. No. ADS-0449M1, Jiangsu Sumeike Biological Technology) according to the manufacturer’s instructions.

### 2.10 RNA extraction and real-time quantitative PCR

Total RNA was extracted from AML-12 cells or liver tissue using the TRIzol (Cat. No. DP424, Tiangen Biochemical Technology) reagent. cDNA was synthesized using the RevertAid First Strand cDNA Synthesis Kit (Cat. No. K1622, Thermo, USA). Subsequently, 50 ng of each cDNA was amplified as a template, and qPCR was performed using a StepOne Plus device (Art. No. 272008342, Life Technologies, USA) with a SYBR^®^ Premix Ex TaqTM II kit (Cat. No. RR820A, Takara, Japan). All samples were run in triplicate, and β-actin was used as an internal control. Data were quantified using the 2^−ΔΔCt^ relative quantitative method, normalized to β-actin expression, and expressed as the ratio of *Zbtb20* or *Zhx2* to *β-actin* mRNA levels. Primers were designed as follows:

*Zbtb20*-F: 5’-TCTCCAGCCCCAGCCCTC-3’,
*Zbtb20*-R: 5’-GAAGGTTGATGCTGTGAATGCG-3’;
*Zhx2*-F: 5’-TCAAAGAGAAAAGCCAAGGACAG-3’,
*Zhx2*-R: 5’-CAAGTCGGGATAGAGCACCATT-3’;
*β-actin*-F: 5’-AGGTCATCACTATTGGCAACGAG-3’,
*β-actin*-R: 5’-TTGGCATAGAGGTCTTTACGGAT-3’.

### 2.11 Chronic liver injury mouse model construction

Wild-type C57BL/6 mice (male, 6–8 weeks) were purchased from Vital River Laboratories (Beijing, China) and were housed in a clean, temperature-controlled environment with a 12-hour cycle of light and darkness, with direct access to regular laboratory food and water. The mice were injected intraperitoneally with 20% CCl4 or corn oil (Cat. No. 032-17016, BioDee, China) twice a week at a dose of 2 mg/kg (18). The mice were euthanized by anesthesia overdose at weeks 3/6/10 of modeling. Blood samples and liver tissue were collected for further analysis. The experimental protocols of this work followed the guidelines and regulations of our Ethics Committee and were approved by the Committee of Animal Use for Research and Teaching at The Air Force Medical Center.

### 2.12 Serum aspartate aminotransferase and alanine aminotransferase assay

To evaluate the hepatic injury at the enzymatic level, serum AST and ALT levels were measured by using an AST (Cat. No. C010-2-1, Nanjing Jincheng, China) or ALT Assay Kit (Cat. No. C009-2-1, Nanjing Jincheng, China) based on manufacturer’s instructions.

### 2.13 Tissue and blood samples processing and histological analysis

Serum samples were collected by centrifugation of whole blood samples at 3000 rpm for 15 min at 4 °C and stored at −80 °C. Liver tissues were fixed in 4% Paraformaldehyde for 72 h, processed histologically, embedded in paraffin blocks, cut to 4 μm sections and stained with H&E or Picrosirius red staining. Individual scores were calculated as the sum of steatosis (0-3), ballooning (0-2) and lobular inflammation (0-3). Sirius red positive area and lipid droplet area were calculated using a BZ-X800 microscope (Keyence, Osaka, Japan) at 20× magnification.

### 2.14 Western blot

Whole-cell or tissue lysates were separated by SDS-PAGE on denaturing 10% or 12% gels and transferred to polyvinylidene fluoride membranes (Cat. No. ISEQ00010, Millipore, USA) (21). Blots were blocked in 5% milk for 1 h at room temperature and then separately incubated at 4 °C overnight with the following specific primary antibodies: anti-Zbtb20 (Cat. No. sc-515370, Santa Cruz Biotechnology, USA), anti-Zhx2 (Cat. No. ab205532, Abcam, UK) and anti-β-actin (Cat. No. 66009-1-Ig, Proteintech, China). Anti-Zbtb20 and anti-Zhx2 primary antibodies were diluted 1:1000, and anti-β-actin primary antibody was diluted 1:10000. After rinsing, blots were incubated in HRP-conjugated anti-rabbit or anti-mouse (Cat. No. A0239 and A0216, Beyotime, China) secondary antibodies for 1 h at room temperature. Enhanced chemiluminescence (Cat. No. 34094, Thermo, USA) was used to detect immunoreactive bands.

### 2.15 Statistical analysis

SPSS Statistics 26.0 (IBM, USA) and Prism 8.0 (GraphPad, USA) software was used to analyze the data. All data are shown as Means ± SD. Student’s t-test was used to analyze differences between two groups. One-way ANOVA was used to compare three or more groups. Significant differences are expressed in the figures and figure legends as *P < 0.05. All cell experiments were repeated using three biological replicates. Six mice were used for all animal experiments.

## 3 Results

### 3.1 ATAC-seq analysis showed that CCl4 induced the increase of chromatin accessibility in chronic liver injury hepatocytes

**Figure 1:**
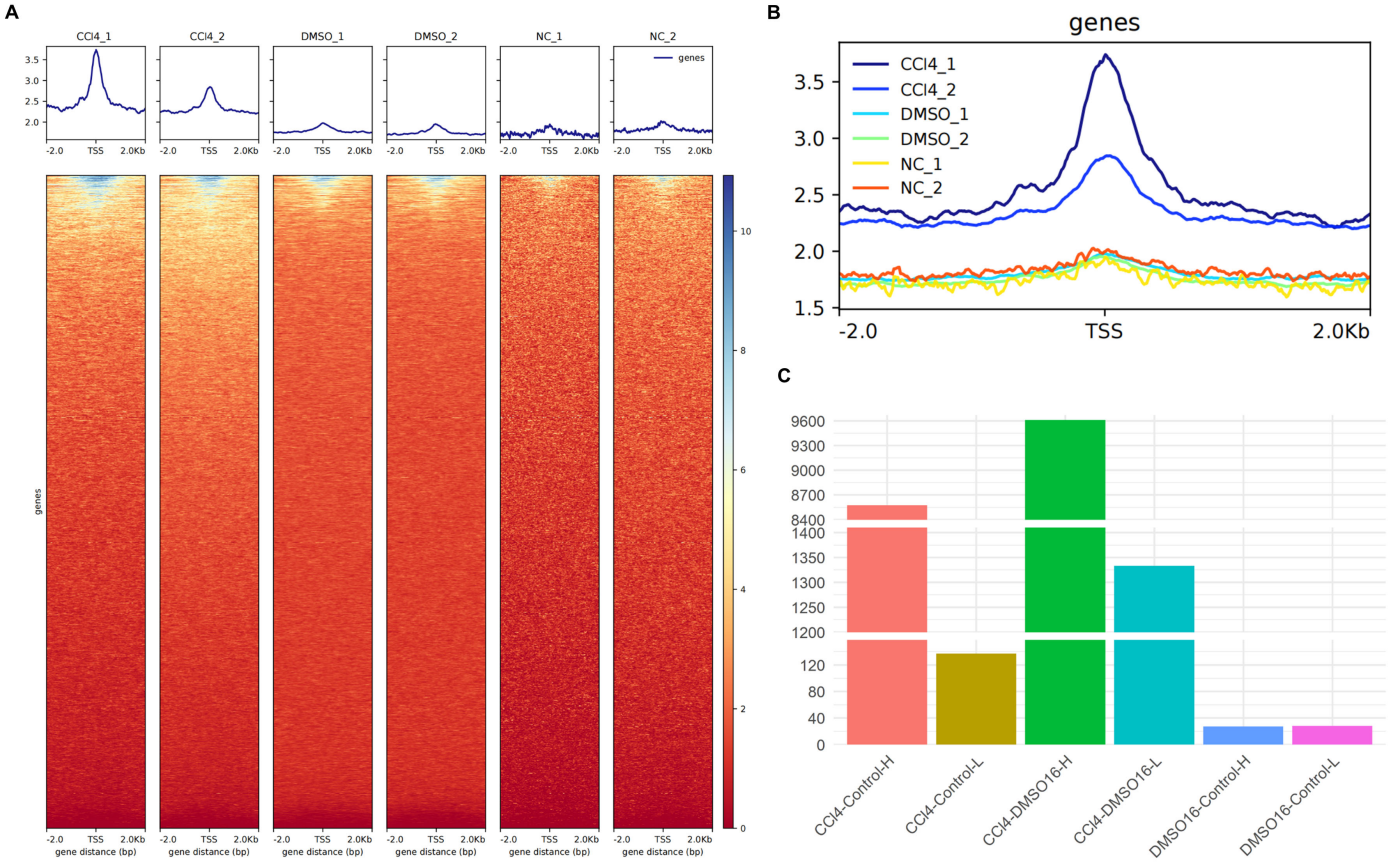
ATAC-seq analysis of chromatin accessibility of CCl4-induced chronic liver injury hepatocytes. **(A)** the enrichment map of ATAC-seq signal around the transcription start site (TSS) in different groups (up) and the enrichment thermogram around TSS (down); **(B)** aggregated enrichment map of ATAC-seq signals around TSS for all groups. **(C)** histogram of the number of genes with peak variation multiple greater than 0 between different groups.

We compared the open peaks between the CCl4-treated group and control groups using ATAC-seq data. The results showed that the chromatin accessibility of hepatocytes treated with CCl4 was significantly higher than that of both the Dimethyl sulfoxide (DMSO)-treated natural recovery group and the untreated control groups (**Figures 1A, B**). Statistical analysis of genes with peak variation multiples revealed that between the CCl4 and control groups, there were 8,573 genes with peak variation multiples greater than 0 (CCl4-Control-H) and 137 genes with peak variation multiples less than 0 (CCl4-Control-L), indicating that the number of genes with increased peaks was much higher than those with decreased peaks. A similar trend was observed between the CCl4 and DMSO groups. However, the number of genes with a peak change multiple greater than 0 and less than 0 was almost equal and less than 40, showing no obvious peak changes between the DMSO and control groups (**Figure 1C**). These results demonstrated that compared to healthy hepatocytes, the chromatin accessibility of hepatocytes treated with CCl4 is significantly increased. Considering the high correlation between chromatin accessibility and histone modifications, we speculate that histone-related regulatory pathways may also be enhanced.

### 3.2 The combined analysis of scRNA-seq and RNA-seq revealed that the activity of histone modification related pathways in hepatocytes increased

**Figure 2:**
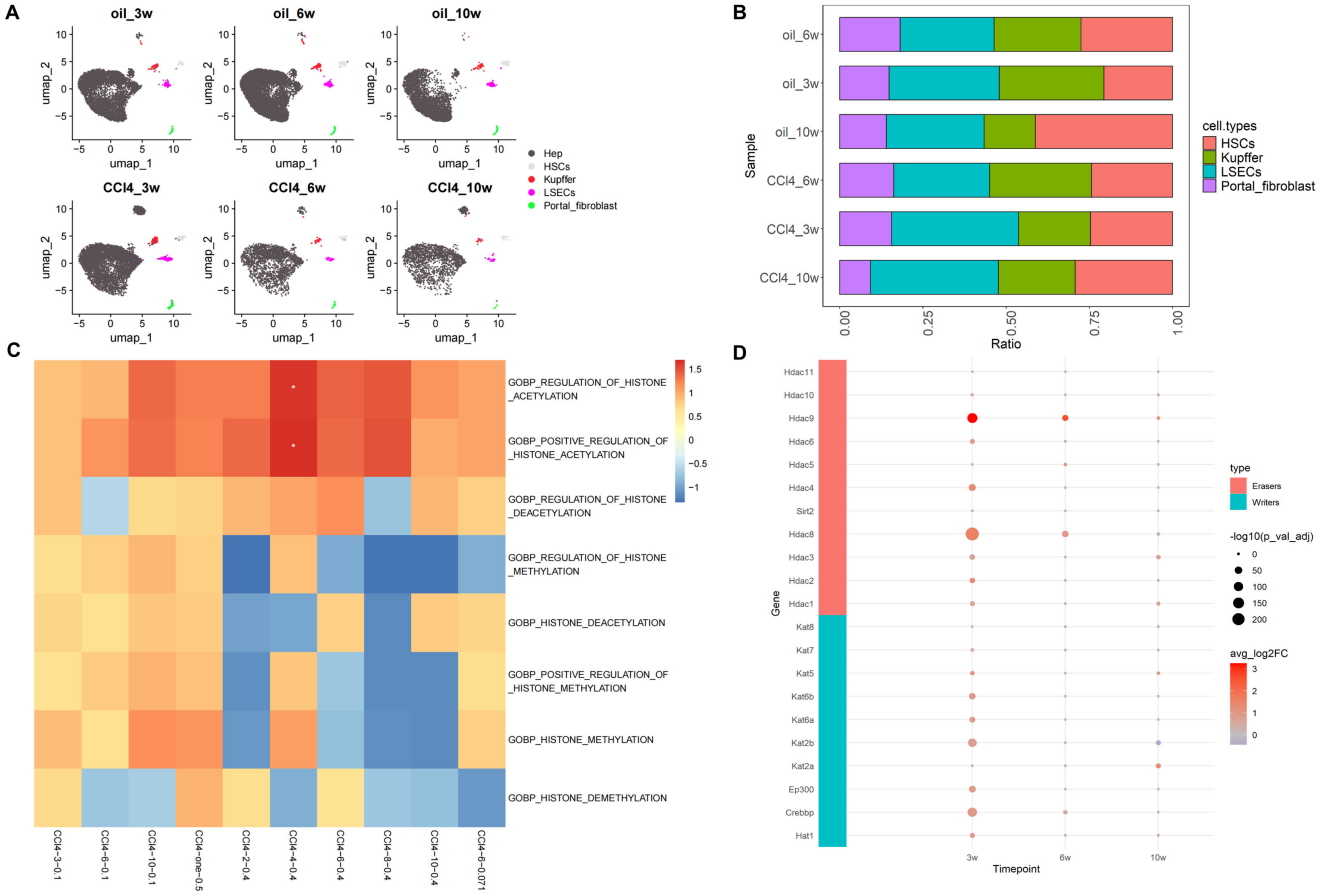
Overview of scRNA-seq and histone-related function analysis. **(A)** Umap distribution of cell subpopulations under CCl4 treatment. **(B)** Histogram of cell proportion change under CCl4 treatment (excluding hepatocytes). **(C)** GO enrichment heatmaps of histone-related pathways. **(D)** Expression of histone acetylation writing and erasing genes.

After processing, the scRNA-seq data from samples subjected to chronic CCl4 stimulation were annotated into five distinct cell subpopulations: hepatocytes, HSCs, Kupffer cells, liver sinusoidal endothelial cells (LSECs), and portal fibroblasts (**Figure 2A**). Given the overwhelming proportion of hepatocytes, these cells were excluded from the histogram depicting cell proportions to better highlight the relative changes in the proportions of the other cell subpopulations. The results of cell proportion indicate that with the prolonging of treatment time, the proportion of hepatocytes and HSCs in the control and stimulation groups showed a slight decrease and increase, respectively (**Figures 2A, B**). Usually, CCl4 mice model was used to study liver fibrosis (characterized by a large increase of HSCs) (22), but the cell proportion in these samples of scRNA-seq did not show such a significant change. Additionally, considering the potential impact of using data from fibrotic samples on the results, analysis of HSCs using fibrosis markers Col1a1 and Col1a2 demonstrated a progressive worsening of liver fibrosis over time (**Supplementary Figure S1**), indicating that the samples had not yet developed fibrosis. Therefore, we considered this dataset could be used for our research on chronic liver injury.

Based on the analysis results of ATAC-seq, we first examined the GO enrichment results of histone-related pathways such as histone acetylation, histone deacetylation, histone methylation, and histone demethylation in scRNA-seq and RNA-seq datasets GSE222576 and GSE207855. In the scRNA-seq data (CCl4-3/6/10-0.1), with the extension of injury time, the NES related to histone acetylation and histone methylation gradually increased, among which the NES related to histone acetylation increased relatively more. In the high concentration group of normal transcription group (CCl4-2/4/6/8/10-0.4), the NES of histone acetylation and methylation pathways showed a trend of increasing first and then decreasing, with the highest value at 4 weeks. To avoid the influence of different sample sources in scRNA-seq and RNA-seq datasets, we also employed a low-concentration RNA-seq dataset (CCl4-6-0.071). The results revealed that the NES of acetylation in the low-concentration CCl4 injection group was higher than that of methylation at 6 weeks, but lower than that in the scRNA-seq data at 6 weeks (CCl4-6-0.1). We also explored common RNA-seq data (CCl4-one-0.5) from a high-concentration acute stimulus, and the results showed that the NES of histone acetylation and methylation pathways was relatively high and enriched (**Figure 2C**).

In order to further analyze the changes of chromatin opening state during hepatocyte injury, we mainly paid attention to “gobp_positive_regulation_of_histone_acetylation” and “gobp_histone_methylation” in enrichment analysis. We investigated the differential expression of 13 core genes associated with the “gobp_positive_regulation_of_histone_acetylation” using the RNA-seq dataset. Among these, only the gene *App* showed significant differential expression with a logFC value greater than 0. “gobp_histone_methylation” included 55 core genes, but differential expression analysis revealed no significantly DEGs.

We also analyzed the differential gene expression in hepatocytes using scRNA-seq data. Among the 13 core genes enriched in the “gobp_positive_regulation_of_histone_acetylation”, nine genes (*App*, *Auts2*, *Baz1b*, *Brca1*, *Dek*, *Ep300*, *Ercc6*, *Sf3b1*, *Smarca5*) were significantly up-regulated in the CCl4-3-0.1 group (p_val_adj < 0.05, log2FC > 0). In the CCl4-6-0.1 group, four genes (*App*, *Auts2*, *Ddx21*, *Ercc6*) showed significant differential expression (p_val_adj < 0.05), with *Ddx21* and *Ercc6* being significantly down-regulated (Log2FC < 0). In the CCl4-10-0.1 group, six genes (*App*, *Auts2*, *Baz1b*, *Ddx21*, *Mybbp1a*, *Sf3b1*) were significantly differentially expressed, with only *Sf3b1* being significantly down-regulated (**Table 1**). Based on these results, we observed that genes involved in histone acetylation exhibited significant changes at different stages of liver damage caused by low-concentration drugs. At 3 weeks, *Auts2* had the most significant p-value (3.24E-149), and *Sf3b1* had the highest (pct.1 = 0.588) proportion of cells. At 6 weeks, *Auts2* again had the most significant p-value (1.79E-31), while at 10 weeks, *Mybbp1a* had the most significant p-value (1.54E-66) and there was a large change in the proportion of *Mybbp1a* with a lower proportion of *Sf3b1* (pct.1 = 0.464) (**Table 1**). Additionally, we analyzed the differential expression of 55 genes related to methylation in scRNA-seq data. The results showed that *Kansl1* had the most significant difference at 3 weeks and the largest proportion of cells, *Xbp1* had the most significant p-value at 6 weeks with a larger proportion of *Gcgr*, and *Setd7* had the most significant difference at 10 weeks with a larger proportion of *Kansl1* (**Supplementary Table S1**).

**Table 1:** Differential expression analysis of histone acetylation genes in scRNA-seq.

Group	Gene	avg_log2FC	pct.1	pct.2	p_val_adj
CCl4-3-0.1	*App*	1.751473	0.131	0.044	4.41E-56
	*Auts2*	4.055308	0.142	0.009	3.24E-149
	*Baz1b*	0.936577	0.392	0.231	6.63E-77
	*Brca1*	3.720688	0.063	0.005	3.29E-58
	*Dek*	1.073672	0.126	0.068	7.99E-22
	*Ep300*	0.960347	0.18	0.093	1.90E-36
	*Ercc6*	0.649576	0.093	0.055	8.55E-10
	*Sf3b1*	0.711974	0.588	0.418	2.74E-83
	*Smarca5*	0.988605	0.121	0.06	1.66E-24
CCl4-6-0.1	*App*	1.101667	0.069	0.037	1.44E-06
	*Auts2*	2.943552	0.046	0.009	1.79E-31
	*Ddx21*	-0.89678	0.04	0.088	2.67E-09
	*Ercc6*	-0.37568	0.075	0.114	0.007711
CCl4-10-0.1	*App*	1.814819	0.084	0.028	6.60E-16
	*Auts2*	1.888906	0.048	0.015	1.40E-08
	*Baz1b*	0.543429	0.453	0.349	8.86E-13
	*Ddx21*	1.195571	0.126	0.058	3.52E-14
	*Mybbp1a*	2.04933	0.225	0.063	1.54E-66
	*Sf3b1*	-0.32248	0.464	0.551	0.000146

Furthermore, the expression of histone acetylation writing and erasing genes gradually decreased from high to low as chronic injury progressed. However, unlike at 3 and 6 weeks, the overall expression of acetylation erasure genes was lower than that of writing genes at 10 weeks. This indicated that acetylation in the drug stimulation group was higher than in the control group at 10 weeks, consistent with the increased activity of the histone acetylation pathway observed in our previous enrichment analysis results (**Figure 2D**). In conclusion, in CCl4-induced chronic liver injury cells, histone acetylation-related pathways were more active and hepatocyte chromatin accessibility increased.

### 3.3 Enrichment analyses of scRNA-seq and RNA-seq demonstrated that metabolism-related pathways enriched in hepatocytes

**Figure 3:**
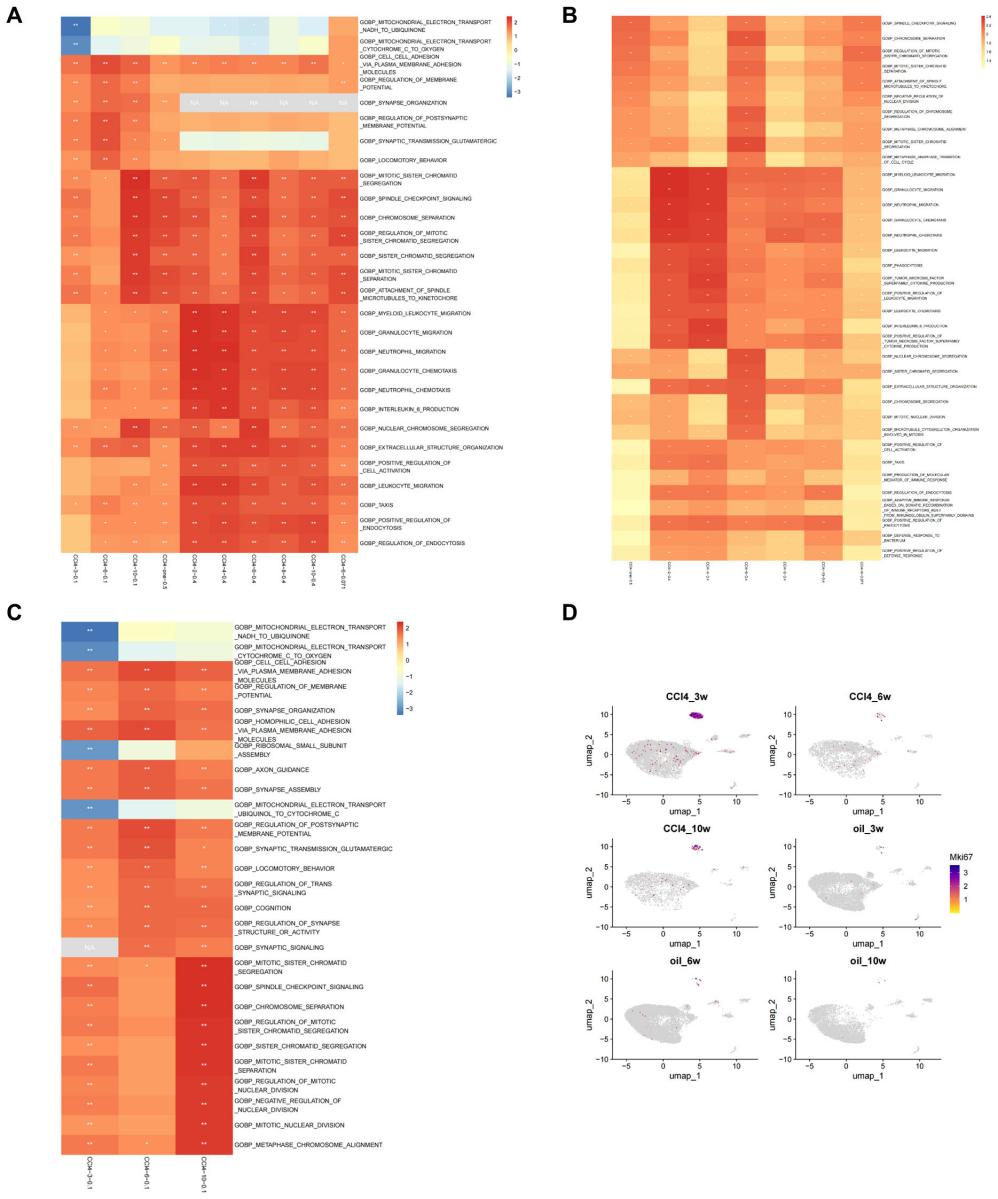
Enrichment analysis of scRNA-seq and RNA-seq. **(A)** Top 5 results of GSEA on scRNA-seq and RNA-seq. **(B)** Top 10 results of GSEA on RNA-seq under different conditions based on GO enrichment. **(C)** Top 10 results of GSEA on scRNA-seq under different conditions based on GO enrichment. **(D)** Umap distribution map of *Mki67* expression in scRNA-seq.

GSEA was performed on hepatocytes under different treatment conditions. The results indicated that the functions of hepatocytes are predominantly associated with cell division, immunity, and inflammation. In the scRNA-seq data (CCl4-3/6/10-0.1), we observed a gradual increase in the NES of inflammation-, immunity-, and mitosis-related pathways with the progression of the injection period. Notably, the NES of the mitosis-related pathway demonstrated a significant elevation at 10 weeks, indicating an elevated level of activity. Additionally, it was evident that the relevance of the pathways associated with inflammation, immunity, and proliferation exhibited a heightened significance when the injection concentration of CCl4 increased (CCl4-2/4/6/8/10-0.4) compared to when the injection concentration was low (CCl4-3/6/10-0.1). The results of low-concentration RNA-seq data (CCl4-6-0.071) showed that the enrichment results of low-concentration CCl4 injection at 6 weeks still indicated that the NES of the division-related pathway was high, and the NES of the immune-related pathway was relatively low, consistent with previous findings. The results of RNA-seq data from high-concentration acute stimulation (CCl4-one-0.5) revealed that the NES of the mitotic pathway was higher, similar to that observed in CCl4-10-0.1 (**Figure 3A**).

We subsequently examined the top 10 enriched results in GSEA of the RNA-seq groups, based on GO enrichment. In the acute group CCl4-one-0.5, the NES associated with chromosome separation was elevated, whereas the immune-related score was lower. In the CCl4-2-0.4 and CCl4-4-0.4 groups, the NESs related to immunity were high, but those associated with chromosome segregation were low. The CCl4-6-0.4 group exhibited increased NES related to chromosome segregation, while the CCl4-8-0.4 and CCl4-10-0.4 groups displayed relatively elevated immune-associated NESs. Furthermore, the chromosome-related NES was higher in the CCl4-6-0.071 group (**Figure 3B**).

GO enrichment analysis of DEGs in RNA-seq revealed that the main enriched functions of DEGs were associated with immunity, cell division, and metabolism. In the acute CCl4-one-0.5 group, chromosome separation and cell cycle regulation were the primary terms. The CCl4-2-0.4 group showed a stronger correlation with metabolic processes, while immune system function had a relatively weaker association. In the CCl4-4-0.4 group, immune correlation enrichment increased, whereas metabolic correlation enrichment decreased. In the CCl4-6-0.4 group, metabolism, immunity, chromosome segregation, and cell cycle all appeared as significant terms. In the CCl4-8-0.4 group, the main functions were metabolism and stimulation, with few terms related to immunity. In the CCl4-10-0.4 group, metabolism was the main function, with minor roles for stimulation and immunity. In the CCl4-6-0.071 group, chromosome separation and cell cycle regulation were the primary terms, with some involvement of immune reactions. Based on GO enrichment results, metabolism is required in the process of chromosome separation and immune responses (**Supplementary Figure S2A**).

Subsequently, we examined the top 10 results of GSEA on scRNA-seq data. From the GSEA results, the NES for mitochondrial electron transfer and ribosome small subunit assembly at 3 weeks was less than 0. The regulation of membrane potential and synaptic signal transmission reached their highest levels at 6 weeks, with NES decreasing at 10 weeks. In pathways related to chromosome segregation, the NES was greater than 0 and significant at 3 weeks, low and insignificant at 6 weeks, and the highest and significant at 10 weeks (**Figure 3C**). We then performed GO enrichment analysis on the DEGs from scRNA-seq data, revealing that the top 5 terms primarily focused on cell division and metabolism (**Supplementary Figure S2B**). We employed the cell proliferation marker *Mki67* to analyze proliferation in cell subpopulations, finding that proliferation occurred exclusively in hepatocytes. An examination of various stages of hepatocyte injury revealed that *Mki67* expression was higher at 3 weeks, lower at 6 weeks, and exhibited an upward trend at 10 weeks. These findings were consistent with the trajectory of change observed in the NES of chromosome segregation-related signaling pathways (**Figure 3D**).

Combining the above results, the open genes appear to be primarily located in those associated with mitotic proliferation and metabolism of hepatocytes. The metabolic process plays a crucial role in CCl4-induced chronic liver injury, particularly focusing on FA metabolism. This is evidenced by the enrichment results from both overall liver tissues and hepatocytes specifically.

### 3.4 Expression of key genes related to FA transport, activation, *de novo* synthesis and oxidation

**Figure 4:**
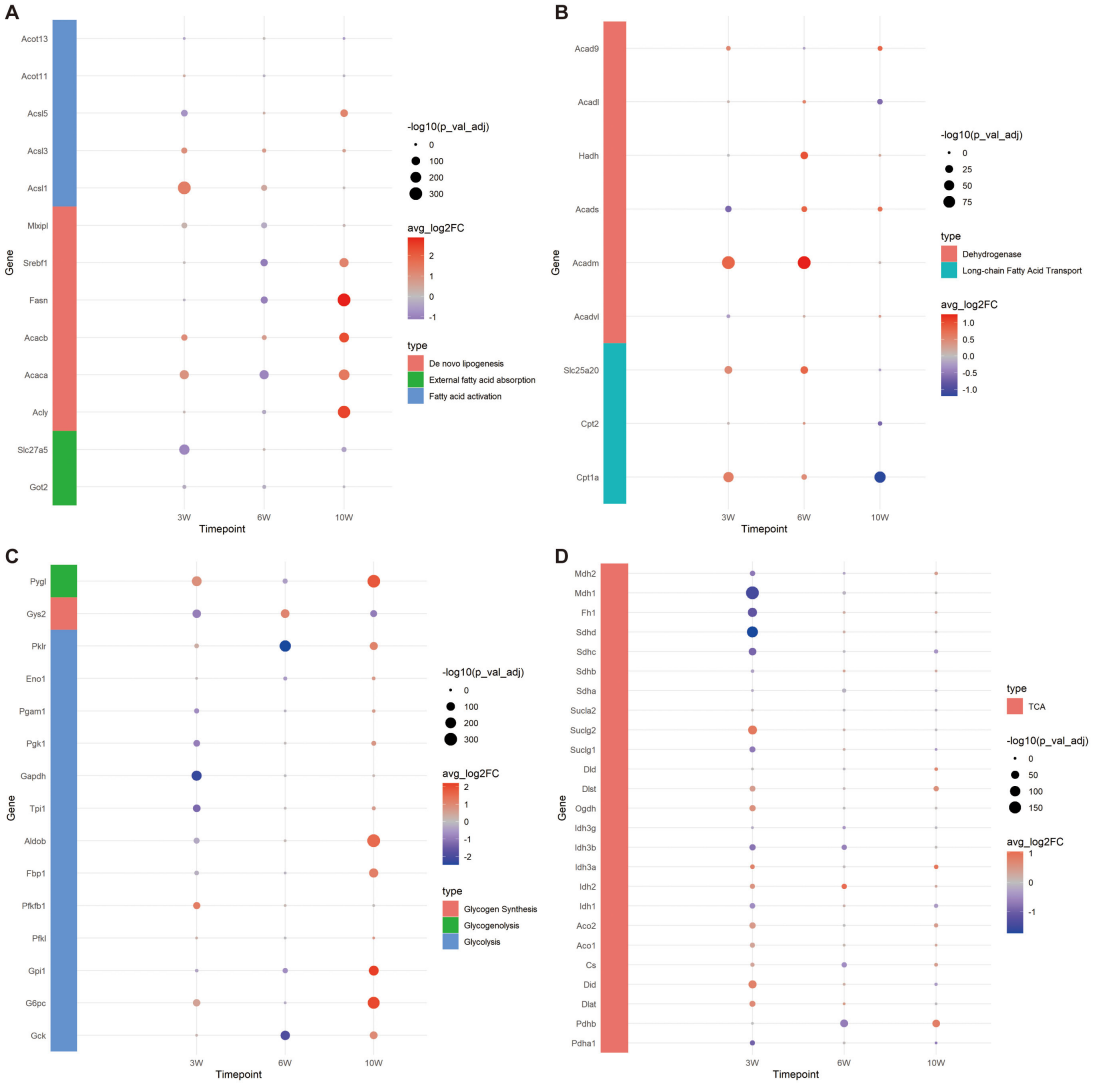
Expression of genes related to FA and glucose metabolism. **(A)** Bubble plot of gene expression related to FA external absorption, activation and *de novo* synthesis. **(B)** Bubble plot of FA transport and oxidation related gene expression. **(C)** Bubble plot of gene expression related to glycolysis, glycogen synthesis and decomposition. **(D)** Bubble plot of tricarboxylic acid (TCA) cycle related gene expression.

Based on the common pathways of FA metabolism, we first explored whether the capacity for external FA absorption was enhanced. We investigated the expression levels of *Got2* and *Slc27a5*, two key genes involved in FA uptake. At 3 weeks, the expressions of both *Got2* and *Slc27a5* were significantly lower in the injury group compared to controls. By 6 weeks, the expression of *Got2* in the injury group had significantly decreased, while by 10 weeks, the expression of *Slc27a5* in the injury group had notably reduced (**Figure 4A**). These results suggest that the liver’s ability to absorb external FAs diminishes during CCl4-induced chronic injury.

To investigate whether the capacity for internal *de novo* FA synthesis is augmented, we assessed the expression levels of key genes involved in this pathway: *Acly*, *Acaca*, *Acacb*, *Fasn*, *Srebf1* and *Mlxipl*. Our findings revealed that at 3 weeks, the expression levels of *Acaca*, *Acacb*, and *Mlxipl* were notably elevated in the injured group, suggesting an enhanced capability of hepatocytes to convert acetyl-CoA into malonyl-CoA through carboxylation. By 6 weeks, with the exception of *Acacb*, the expression of the remaining genes in the injured group diminished, implying a reduced FA synthetic potential. Remarkably, at 10 weeks, aside from *Mlxipl*, there was a substantial upregulation in the expression of other genes within the injured cohort, highlighting that the peak period for *de novo* FA synthesis occurs at this time point (**Figure 4A**).

To examine the alterations in FA activation capabilities within cells, we conducted an analysis of gene expression for key enzymes involved in this process: *Acsl1*, *Acsl3*, *Acsl5*, *Acot11*, and *Acot13*. At the 3-week, a significant decrease was observed in the expression of *Acsl5* within the injured group. Conversely, there was a marked upregulation in the expression of both *Acsl1* and *Acsl3*. By week 6, *Acsl1* and *Acsl3* in the injury group exhibited an elevated expression. At the 10-week, a notable increase in the expression of both *Acsl3* and *Acsl5* was observed in the injury group. Throughout this chronic injury timeline, the expression patterns of *Acot11* and *Acot13* remained constant, implying no significant fluctuations in the conversion of acyl-CoA back to free FAs. Notably, the reduced expression of *Acsl5* at 3 weeks in the injured group could potentially be interpreted as a compensatory mechanism in response to the heightened expression of *Acsl1*. Collectively, these results underscore the robust capacity for FA transformation into fatty acyl-CoA throughout the entire observation period (**Figure 4A**).

In the process of FA oxidation, carnitine palmitoyl transferase I (*Cpt1a*) play a pivotal role by acting on long-chain acyl-CoA, converting it into acylcarnitine, and facilitating its transport into the mitochondria. Our study revealed that the expression level of *Cpt1a* was elevated in the injury group at both 3 and 6 weeks, but exhibited a decrease by week 10. The expression of the carnitine/acylcarnitine translocase *Slc25a20* was found to be notably increased in the injury group at 3 and 6 weeks, followed by a reduction at week 10. *Cpt2*, which is essential for the conversion of acylcarnitine back to acyl-CoA within the mitochondrial matrix to initiate β-oxidation, showed no significant difference compared to the control group at 3 and 6 weeks; however, its expression was lower in the injury group by week 10. These findings suggest that the capacity for FA oxidation transportation is robust at 3 and 6 weeks under chronic CCl4 stimulation but diminishes by week 10 (**Figure 4B**).

Furthermore, we investigated the expression profiles of long-chain, medium-chain, and short-chain FA dehydrogenases, which are primarily involved in β-oxidation. Results suggest that the capacity for FA β-oxidation is most pronounced during the initial and intermediate stages, peaking at 6 weeks, followed by a decline at 10 weeks. This temporal pattern corresponds with the observed fluctuations in mitochondrial transport efficiency related to FA oxidation (**Figure 4B**).

Overall, our findings indicate that the hepatocytes’ capacity to absorb external FAs diminished across all stages following CCl4-induced chronic injury, while their ability to synthesize FAs exhibited a slight increase at 3 weeks, followed by a decrease at 6 weeks and a significant enhancement by 10 weeks. Acetyl-CoA generation through FA oxidation persisted throughout all stages examined.

### 3.5 The expression of genes related to glycolysis, glycogen synthesis, decomposition and tricarboxylic acid cycle

Based on the aforementioned analysis, we observed that the expression levels of *Srebf1* and *Mlxipl* were diminished at 6 weeks, indicating a reduced capacity for lipid production and carbohydrate metabolism. Further investigation was conducted on the expression of genes involved in glycolysis, glycogen synthesis, decomposition, and TCA cycle. At 3 weeks, no significant difference was detected in the expression of *Gck*. However, notable variations were evident in the expressions of *Pygl*, *G6pc*, *Pfkfb1*, and *Pklr*, which were significantly elevated in the injury group. This suggests an enhancement in glycogen hydrolysis and glucose conversion efficiency, as well as an increased capability to convert phosphoenolpyruvate into pyruvate (regulated by *Pklr*). Conversely, the expressions of *Gys2*, *Gpil*, *Aldob*, *Tpi1*, *Gapdh*, *Pgk1* and *Pgam1* were substantially decreased in the injury group, implying inhibited glycolysis and reduced glycogen synthesis. By 6 weeks, there was a significant downregulation of *Gck* expression in the injury group, with most glycolysis-related genes exhibiting lower expression levels; however, glycogen synthesis potential appeared to strengthen while hydrolysis weakened. At 10 weeks, genes associated with glycogen hydrolysis and glycolysis were highly expressed, except for those related to glycogen synthesis, which indicated a weakening ability. These findings collectively suggest that glycolysis became more prominent at 10 weeks (**Figure 4C**).

Following glycolysis, glucose can be further metabolized into FAs through the TCA cycle via *de novo* lipogenesis (23). Consequently, we investigated the expression of genes associated with the TCA cycle (**Figure 4D**). At 3 weeks, reactions within the TCA cycle were enhanced, as evidenced by increased expression levels of *Dlst*, *Ogdh*, *Idh*, *Aco*, *Cs*, *Did* and *Dlat*. Conversely, the expression of succinate dehydrogenase (*Sdh*), which is involved in the conversion of succinate to fumarate, was decreased, along with a reduction in the expression of downstream enzymes *Fh* and *Mdh*, indicating diminished activity in subsequent reactions. The expression of the succinate transporter *Sucl* family remained unchanged, suggesting potential impacts on mitochondrial ETC function. By 6 weeks, the TCA cycle exhibited a suppressed state, whereas at 10 weeks, it was not significantly inhibited.

In summary, the capacity for carbohydrate utilization in hepatocytes chronically damaged by CCl4 progressively increased over time, potentially correlating with enhanced *de novo* FA synthesis observed at 10 weeks post-injury.

### 3.6 Expression and function prediction of ETC related genes and the expression of *Zhx2*

**Figure 5:**
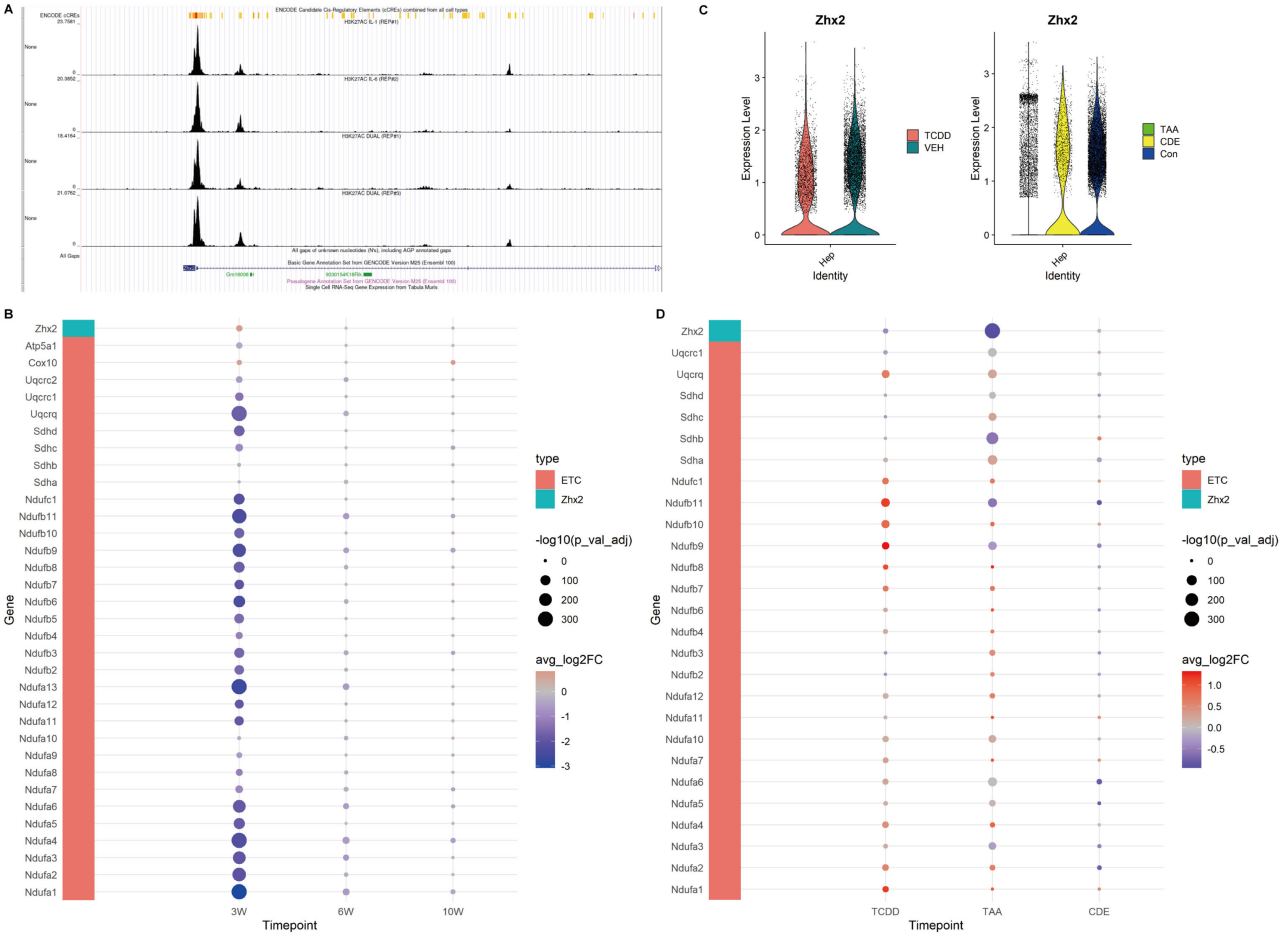
Expression of *Zhx2* and ETC related genes in CCl4-induced chronic liver injury and chronic injury of TCDD, TAA and CDE. **(A)** Chip-seq tracks of H3K27ac on *Zhx2* in hepatocytes. **(B)** *Zhx2* and ETC related gene expression bubble plot. **(C)** *Zhx2* expression in chronic injury of TCDD, TAA and CDE violin plot. **(D)** *Zhx2* and ETC related gene expression bubble plot in chronic injury of TCDD, TAA and CDE.

Based on the aforementioned results, we further employed GSEA to examine the expression profiles of genes associated with the ETC. Our findings confirmed that the ETC was indeed inhibited at 3 weeks, as evidenced by reduced expression levels of ETC-related genes in the injured group. However, at 6 and 10 weeks, the expressions of these genes gradually recovered over time within the injured group (**Figure 5B**).

When integrated with our analysis of glucose metabolism, it was observed that the TCA cycle was enhanced up until the succinate stage but subsequently decelerated. Concurrently, both glycolysis and the ETC were found to be suppressed, suggesting the presence of alternative metabolic substrates supporting the TCA cycle. To investigate this possibility, we analyzed amino acid metabolism and discovered that genes involved in branched-chain amino acid metabolism and glutamine metabolism were significantly upregulated in the injured group at 3 weeks (**Table 2**). These findings imply that the TCA cycle may be sustained, at least partially, through metabolites derived from amino acid catabolism.

**Table 2:** Differential expression analysis of genes related to branched-chain amino acid metabolism and glutamine metabolism.

Time	Genes	log2FC	p_val_adj
3w	*Gls*	2.554404	8.55E-17
	*Glud1*	0.465153	3.82E-42
	*Bcat1*	5.301665	8.18E-12
	*Bcat2*	1.556717	1.90E-116
	*Bckdhb*	0.982579	9.15E-168
	*Dbt*	0.588261	4.06E-05
	*Got1*	1.075548	7.45E-164
	*Got2*	-0.26727	5.00E-05
6w	*Glud1*	-0.47274	3.16E-42
	*Bcat2*	-0.62184	1.63E-06
	*Bckdhb*	0.47919	4.43E-13
	*Dbt*	1.211602	4.37E-30
	*Got1*	0.818492	2.81E-44
	*Got2*	-0.25649	0.000264
10w	*Bckdha*	-0.99312	5.37E-31
	*Dld*	0.714332	0.076287
	*Got1*	1.050561	2.69E-40

We identified *Zhx2* as a key regulatory gene in the upstream regulation of the ETC based on literature review. Our analysis revealed that its expression was elevated at 3 weeks. Further exploration using the Cistrome database demonstrated that, within the Chip-seq of H3K27ac in hepatocytes, the peak intensity at the *Zhx2* promoter region was notably higher, suggesting a propensity for acetylation. However, at both 6 and 10 weeks, no significant differences in *Zhx2* expression were observed (**Figures 5A, B**).

These findings collectively suggest that during the early stages of chronic CCl4-induced liver injury, the mitochondrial ETC is inhibited. As the injury progresses and recovery mechanisms engage, the functionality of the ETC and oxidative phosphorylation gradually approach a near-normal state.

### 3.7 Expression of ETC related genes and *Zhx2* in other chronic liver injury

In order to compare with other chronic liver injuries, we examined the expression levels of ETC-related genes and *Zhx2* in liver injuries induced by TCDD, TAA and CDE. The results revealed that in chronic injuries caused by both TCDD and TAA, *Zhx2* expression was diminished in the injured groups, whereas the expression of ETC-related genes was elevated. In the data of chronic injury induced by CDE, no significant difference was observed in *Zhx2* expression, and the expression patterns of different ETC-related genes exhibited inconsistencies (**Figures 5C, D**). Collectively, these data suggest that while *Zhx2* expression varies under the influence of different drugs, there exists a consistent negative correlation between the expression of *Zhx2* and that of ETC-related genes, aligning with our previous findings.

### 3.8 Comparison of cellular functions of chronic liver injury in different periods

**Figure 6:**
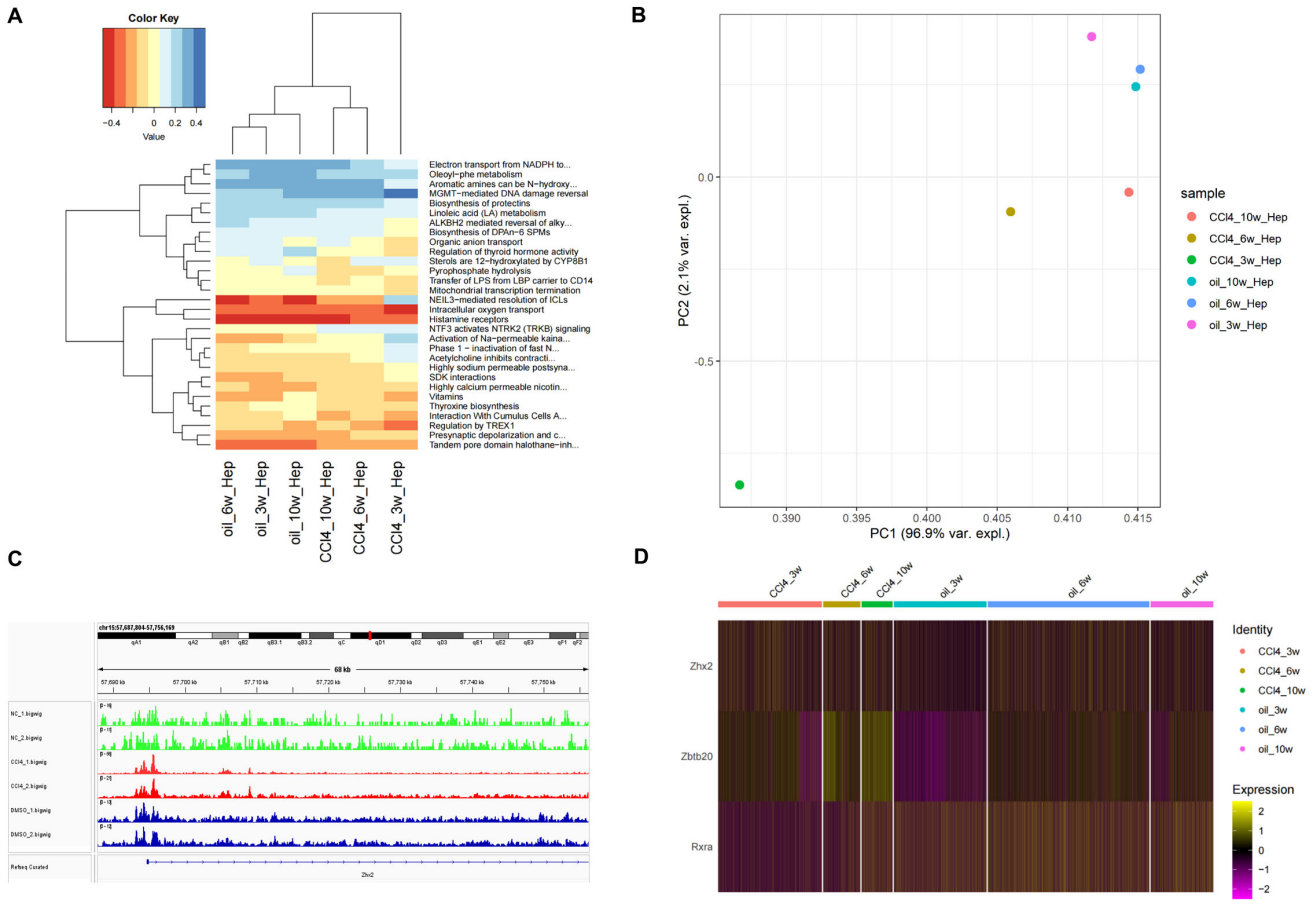
Analysis of cell function and transcription regulatory factors of *Zhx2* in different periods. **(A)** Heatmap of top 30 pathways of chronic liver injury cells in different periods. **(B)** PCA diagram of CCl_4_-induced chronic liver injury cells in different periods. **(C)** ATAC-seq tracks for *Zhx2* in six ATAC-seq data. **(D)** Expression heatmap of *Zhx2*, *Zbtb20* and *Rxra* in scRNA-seq data.

The heat map of the top 30 pathways revealed a significant difference between the CCl4-3w and the control group. In contrast, both the CCl4-6w and CCl4-10w groups exhibited functional profiles closer to that of the control group, indicating a recovery trend over time (**Figure 6A**). Principal Component Analysis (PCA) further corroborated these findings; PC1 demonstrated that CCl4-3w was markedly distinct from the other groups in terms of function. By CCl4-6w, the function state began to normalize, with CCl4-10w showing even closer resemblance to normal function (**Figure 6B**). These results collectively support the hypothesis that metabolism in chronically injured hepatocytes transitions from an initial inhibitory phase towards a state of metabolic balance as recovery progresses.

### 3.9 TFs regulating *Zhx2*

A significant difference was observed in the expression of *Zhx2* at 3 weeks post-treatment, yet no substantial differences were noted at 6 and 10 weeks (**Figure 6D**). Subsequent analysis of ATAC-seq data revealed that the peak value of *Zhx2* (CCl4_1/2.bigwig) following CCl4 administration was notably higher compared to the control group, suggesting an enhancement in chromatin accessibility. However, its expression exhibited a gradual decline with increasing duration of injections (**Figure 6C**).

The increase in *Zhx2* chromatin accessibility, coupled with the subsequent inhibition of its expression over time, suggested the potential involvement of multiple TFs interacting with *Zhx2*. To investigate this further, we compared scRNA-seq data between CCl4-6w and CCl4-3w, as well as between CCl4-10w and CCl4-3w. The differential expression analysis was conducted with a significance threshold set at padj < 0.05 and logFC > 0. We then intersected the resulting DEGs with those identified in the control versus CCl4 comparison to refine our list. Finally, we cross-referenced this refined list with a database of known TFs to identify ten candidate TFs: *Zbtb20*, *Tef*, *Mafb*, *Rxra*, *Egr1*, *Xbp1*, *Ppara*, *Ncor2*, *Zbtb7a*, and *Zbtb7c*. We subsequently examined the Cistrome database to determine if these TFs had binding sites to *Zhx2*.

Among the TFs investigated, *Zbtb20*, *Xbp1*, and *Zbtb7a* exhibited peaks at the TSS of *Zhx2*, with *Zbtb20* showing the highest peak. The mouse dataset was utilized for *Xbp1*, while human data were used for the other two TFs. Additionally, *Mafb*, *Rxra*, *Ppara*, and *Ncor2* displayed peaks within the transcription region of *Zhx2*, with *Mafb* demonstrating the highest peak. For these four TFs, mouse data were employed. The human data for *Egr1* indicated an inconspicuous peak value at *Zhx2*. Furthermore, Cistrome lacked data for *Tef* and *Zbtb7c*. In summary, *Zbtb20*, *Mafb*, *Rxra*, *Xbp1*, *Ppara*, *Ncor2*, and *Zbtb7a* are likely regulators of *Zhx2* expression. The binding sites of *Zbtb20*, *Xbp1*, and *Zbtb7a* were located at the TSS of *Zhx2*, while the binding sites of *Mafb*, *Rxra*, *Ppara*, and *Ncor2* were situated within the transcription region (**Supplementary Figure S3**).

We observed that *Zbtb20* and *Rxra* exhibited relatively high peak values, suggesting a significant inhibitory effect on transcription. The expression of *Zbtb20* was consistently up-regulated in the CCl4-treated injury group, with the up-regulation trend intensifying over time. Conversely, the expression of *Rxra* initially decreased in the injured group following CCl4 treatment but gradually recovered as time progressed (**Figure 6D**). By integrating the ATAC-seq results with *Zhx2* expression data, we hypothesize that *Zbtb20* may act as a crucial upstream transcriptional repressor, binding to the TSS of *Zhx2*, which is negatively correlated with the expression pattern of *Zhx2*.

### 3.10 CCl4 increases acetyl-CoA, Zbtb20 and Zhx2 levels both *in vivo* and *in vitro*

**Figure 7:**
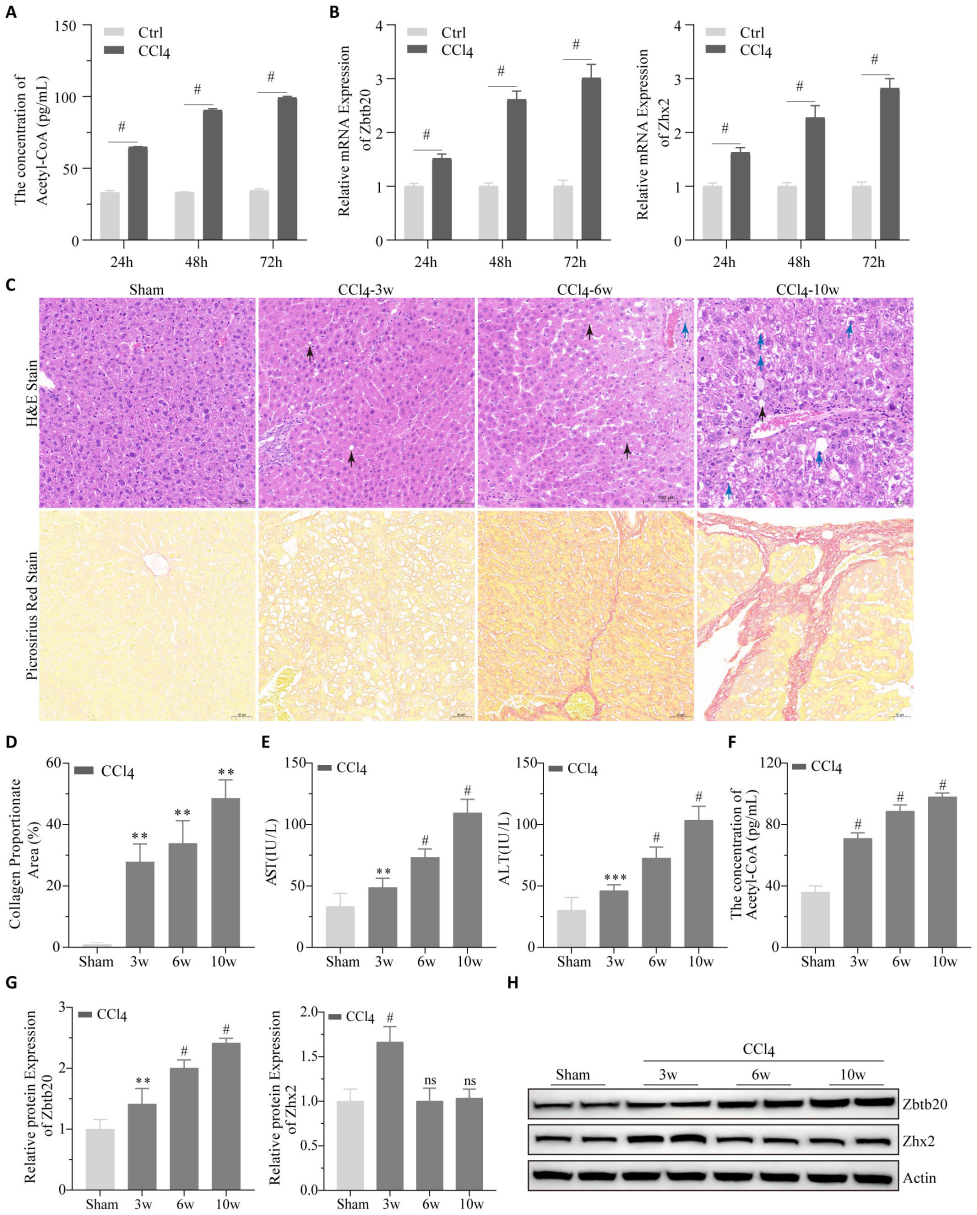
Cell and animal experiments. **(A)** Acetyl-CoA were detected by ELISA and **(B)** *Zbtb20* and *Zhx2* mRNA were detected by qPCR after CCl4 treatment of AML-12 cells for 24h, 48h and 72h, respectively. **(C)** Representative images of H&E staining (upper panel) and Picrosirius red staining (lower panel) of paraffin sections of liver tissues in mice by intraperitoneal injection of CCl4 or solvents 3, 6, and 10 weeks later. Black arrows indicate cells with intracytoplasmic vacuoles, and blue arrows indicate cells with pale and edematous cytoplasm. **(D)** Statistical plots of collagen proportionate area in Picrosirius red staining against C graph. AST and ALT **(E)** acetyl-CoA **(F)** Quantification data **(G)** and representative Western blot images **(H)** for *Zbtb20* and *Zhx2* in C57 mice by intraperitoneal injection of CCl4 or solvents after 3, 6, and 10 weeks. n=6. ** p<0.01, *** p<0.001, # p<0.0001, ns indicates no statistical significance.

To validate our bioinformatic results, changes in acetyl-CoA, *Zbtb20* and *Zhx2* were first detected after CCl4 treatment of AML12 cells; as shown in **Figures 7A, B**, CCl4 treatment significantly increased their levels in a time-dependent manner. Subsequently, the chronic liver injury mouse models were established by intraperitoneal injection of CCl4. The degree of liver tissue structure abnormality increased with the increase of CCl4 treatment time; steatosis, the number of hepatocytes with intracytoplasmic vacuoles and edema increased (**Figure 7C**). Pathological indices of the liver such as collagen proportionate area (**Figure 7D**), AST and ALT (**Figure 7E**) also worsened with increasing CCl4-treatment time. We detected changes in acetyl-CoA, Zbtb20 and Zhx2 levels in the chronic liver injury mouse models. Similar to the cellular results, acetyl-CoA (**Figure 7F**), and Zbtb20 (**Figures 7G, H**) continued to rise with increasing treatment time. However, Zhx2 (**Figures 7G, H**) showed an increase in expression only in the week 3, and the expression returned to the normal level after the week 6, consisting with our bioinformatic analysis.

## 4 Discussion

A retrospective analysis conducted on the Chinese mainland reveals that over half of DILI cases involve hepatocyte damage, with an estimated annual incidence exceeding 2.38% (24). However, there remains a paucity of specific biomarkers for DILI (25). CCl4 serves as a prevalent agent for inducing chronic liver injury models, capable of triggering endogenous DILI (26).

In our investigation, we employed datasets from ATAC-seq, scRNA-seq, and RNA-seq analyses of CCl4-induced chronic liver injury cells to elucidate the metabolic homeostasis mechanisms and identify key TFs governing liver cell responses during chronic insult. Subsequently, experimental validation was undertaken using AML-12 cells and mice subjected to CCl4 treatment. We specifically assessed the expression levels of acetyl-CoA, *Zhx2*, and *Zbtb20*, thereby laying a foundational basis for deepening our understanding of DILI and unraveling its underlying pathogenic mechanisms. Our study presents three key innovations: First, this study demonstrates a dynamic interplay between chromatin accessibility and acetyl-CoA levels mediated by histone acetylation-dependent epigenetic remodeling, with their coupled effects evolving alongside disease progression. Second, we discover that the transcriptional repressor *Zbtb20* acts as a critical suppressor of *Zhx2* expression, forming a regulatory axis that plays a crucial role in liver injury responses and offers a novel molecular insight into hepatic disease progression. Finally, temporal features of hepatocyte metabolic reprogramming reveal a transition from early inhibition of ETC to late-phase functional recovery. This process correlates with stage-specific lipid metabolic patterns.

The results of ATAC-seq and the enrichment analysis of RNA-seq and scRNA-seq data demonstrated that CCl4 stimulation increased chromatin accessibility in hepatocytes. Enrichment analysis under varying treatment conditions highlighted significant alterations in pathways primarily associated with cell mitosis, immunity, and metabolism. Based on the results, it is inferred that genes exhibiting increased chromatin accessibility are predominantly associated with cellular processes such as cell mitosis, proliferation, and metabolism, and FA metabolism plays an important role in chronic drug-induced liver injury.

In healthy human liver, FAs can be oxidized for energy or be transported via VLDL to other organs (27). Hepatocytes are responsible for synthesizing, storing, and secreting lipids to maintain lipid homeostasis, which is essential for the regulation of lipid metabolism (28). Disturbances in lipid metabolism can lead to liver diseases and even cancer (29–33). Therefore, we analyzed the expression of key genes involved in FA transport, activation, *de novo* synthesis, and oxidation. The results indicated that the ability of hepatocytes to absorb external FAs was diminished for an extended period following chronic drug-induced injury (3–10 weeks), while the capacity for FAs to acquire acetyl-CoA increased. The *de novo* synthesis of FAs exhibited a slight increase at 3 weeks, decreased at 6 weeks, and significantly increased at 10 weeks. Notably, the expression of *Srebf1* and *Mlxipl* decreased at 6 weeks. *Srebf1* is a key TF in lipogenesis, which can activate new lipid synthesis and cholesterol uptake, thereby maintaining lipid homeostasis (34). *Mlxipl* (also known as *ChREBP*) is a TF that regulates the lipogenesis of liver tissue and can be activated by carbohydrate metabolites to regulate glycolysis (35). Thus, the ability of lipid production and carbohydrate metabolism was low at 6 weeks.

Within the liver, insulin can either promote or reduce glucose synthesis by activating or downregulating glycogen-related enzymes (36). We further investigated the expression of related genes in glycolysis and TCA cycle. The results revealed that as time progressed, the utilization ability of carbohydrates increased, and the ability of oxidative phosphorylation gradually recovered, which corresponds to the previously mentioned increase in the *de novo* synthesis of FAs at 10 weeks.

The changes in hepatocyte pathways and the PCA analysis of scRNA-seq data under different conditions revealed a strong relationship between the chromatin accessibility of genes and the concentration of acetyl-CoA. Previous study has shown that acetyl-CoA can act as a substrate for histone acetylation to promote oncogenesis (37). For instance, in a hepatocellular carcinoma model, ACOT12 deficiency-induced acetyl-CoA accumulation specifically increased histone acetylation at the Twist2 locus (38). Our data are consistent with this finding. Notably, although our data demonstrate a correlation between chromatin accessibility and acetyl-CoA concentration, chromatin remodeling is inherently subject to multifactorial regulation, including nucleosomal interactions and DNA methylation modifications (39). The current experimental framework cannot fully exclude potential confounding effects from these coexisting regulatory mechanisms, which may require further mechanistic investigations to disentangle their relative contributions. Additionally, as an important gene in the rate-limiting step of glycolysis, the increased expression of *Pklr* at 3 weeks suggested the presence of metabolites from other sources to support the TCA cycle. Subsequent amino acid metabolism analysis suggested that these metabolites could be the source supporting the TCA cycle. Existing studies have also found that *Pklr* is involved in the regulation of lipid metabolism and mitochondrial activity (40), which may be one reason why the intracellular metabolic response is not completely consistent with that at 6 and 10 weeks after CCl4-induced liver injury.

Combined with the significant increase in FA synthesis ability at 10 weeks observed in previous enrichment analysis, we speculate that lipid accumulation in hepatocytes at this time point may play a certain reparative role. This is consistent with recent research, which demonstrates that the steady state of liver lipid metabolism is crucial for maintaining the regenerative potential of the liver (41).

*Zhx2* is a key regulatory gene in the upstream of the mitochondrial ETC. Knockout of *Zhx2* can promote the repair of acute liver injury by regulating mitochondrial function (20). Furthermore, *Zhx2* was found to inhibit *de novo* synthesis of FA and subsequent progression of hepatocellular carcinoma (42). Our research results indicate that the chromatin accessibility of *Zhx2* increases following CCl4 treatment, and *Zhx2* is prone to acetylation. However, during chronic liver injury, the expression of *Zhx2* exhibits a trend of initially increasing and then decreasing, suggesting potential regulation by TFs. Further exploration identified two TFs, *Zbtb20* and *Rxra*, that may regulate the expression of *Zhx2*. *Rxra* can form heterodimers with *Rarb* and act as transcription repressor activator (43). Through analysis, we found that *Zbtb20* has a higher inhibitory effect on the transcription of *Zhx2*, and its expression trend is more consistent with that of *Zhx2*. Knocking down *Zbtb20* promotes liver repair after 70% hepatectomy (44). We speculated that *Zbtb20* may be an important upstream transcription repressor and bind to the transcription initiation site of *Zhx2*. *Zbtb20* restores the equilibrium of hepatocyte energy metabolism and detoxification capacity by suppressing *Zhx2* transcription, thereby alleviating its dual repression on mitochondrial ETC-related genes and sexually dimorphic CYP enzymes (45). This regulatory axis may coordinate ETC efficiency with cytochrome P450-mediated redox homeostasis to temporally balance metabolic suppression and regenerative demands during liver injury repair.

The results in cell and animal experiments verified our conjecture. As an important factor in the metabolic balance mechanism of liver injury stimulated by CCl4, the expression of acetyl-CoA increased with the increase of injury time. Additionally, the expression trend of *Zbtb20* is corresponding, and also increased with the increase of injury time, which is consistent with the research results of Zhang et al. (20).

There are some limitations to our study. The exploration of the regulatory relationship of *Zbtb20* and *Zhx2* and the CCl4-induced metabolic differences were still insufficient. CRISPR-Cas9 can be used to construct hepatocyte-specific *Zbtb20* or *Zhx2* knockout mouse models. Combined with metabolomics and epigenetic analysis, their direct role in metabolic recovery can be verified. In the next stage, Zbtb20 agonists or Zhx2 inhibitors based on animal models will be the focus, and their potential in improving mitochondrial function and liver injury prognosis will be assessed.

## 5 Conclusions

This study elucidates the molecular mechanisms underlying hepatocyte metabolic reprogramming and epigenetic dynamic regulation in hepatocytes during CCl4-induced chronic liver injury through multi-omics analysis. Notably, our findings reveal that prolonged injury duration induces significant elevation of histone acetylation levels and enhanced chromatin accessibility in hepatocytes, a process closely associated with persistent accumulation of acetyl-CoA. Functionally, acetyl-CoA serves dual roles as both a critical intermediate in *de novo* fatty acid synthesis and a driver of histone acetylation-mediated gene regulation, suggesting that acetyl-CoA metabolism can act as a potential therapeutic target for restoring hepatocyte homeostasis. At the transcriptional regulation level, the study uncovers an interaction mechanism between Zhx2 and Zbtb20. Zhx2, a key suppressor of the ETC, exhibits marked upregulation during the early injury phase (3 weeks), exacerbating energy metabolism dysfunction. However, its expression progressively returns to baseline levels with sustained injury (6–10 weeks), paralleling the gradual recovery of ETC functionality. This regulatory pattern demonstrates negative correlation with the sustained upregulation of transcriptional repressor Zbtb20, indicating the central role of the Zbtb20-Zhx2 axis in mitochondrial functional restoration. Therapeutic intervention targeting the Zbtb20-Zhx2 axis may offer new solutions for ameliorating early-stage mitochondrial dysfunction. Further analyses demonstrate stage-specific characteristics of hepatocyte metabolic reprogramming, early injury phases feature partial TCA cycle obstruction and mitochondrial ETC inhibition with predominant fatty acid β-oxidation, while later stages exhibit glycolytic activation and significantly enhanced *de novo* lipogenesis capacity. These findings highlight the necessity for phase-specific therapeutic approaches. Moreover, combined strategies targeting histone acetylation and metabolic intervention may prove more effective in improving prognosis for DILI.

In conclusion, our findings elucidate the process by which metabolic imbalance transitions towards equilibrium in cells following CCl4-induced liver injury. This process may be associated with the acute accumulation of lipids during liver damage repair, accompanied by increased histone acetylation throughout the entire period and inhibition of mitochondrial ETC activity in the early stage of CCl4-induced liver injury. Our research deeply explored the mechanism of the development process of chronic liver injury and metabolic balance induced by CCl4, which supplemented the pathological mechanism of DILI, and provided a new drug development direction for targeted treatment of chronic liver injury.

## Data Availability

The original contributions presented in the study are included in the article/**Supplementary Material**. Further inquiries can be directed to the corresponding authors.
