# Supplementary material for: ScRNA-seq combined with ATAC-seq analysis to explore the metabolic balance mechanism of CCl4-induced liver inflammatory injury
Source: Front Immunol. 2025 Jun 16;16:1600685. doi: 10.3389/fimmu.2025.1600685 (PMC12206626; doi:10.3389/fimmu.2025.1600685)
Supplement: Supplementary file 1 [file DataSheet1.docx]

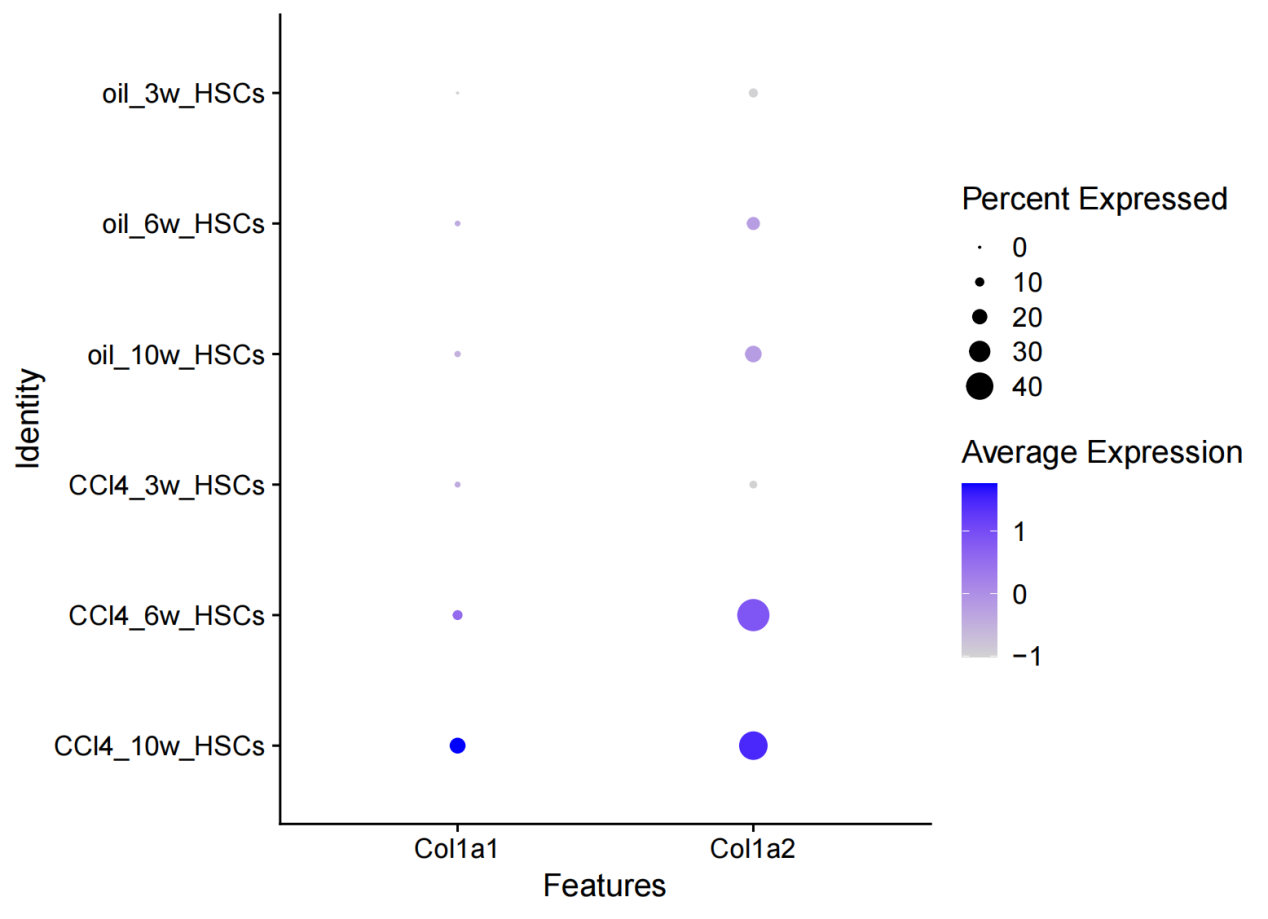


Supplementary Figure 1| Analysis of liver fibrosis marker gene expression.


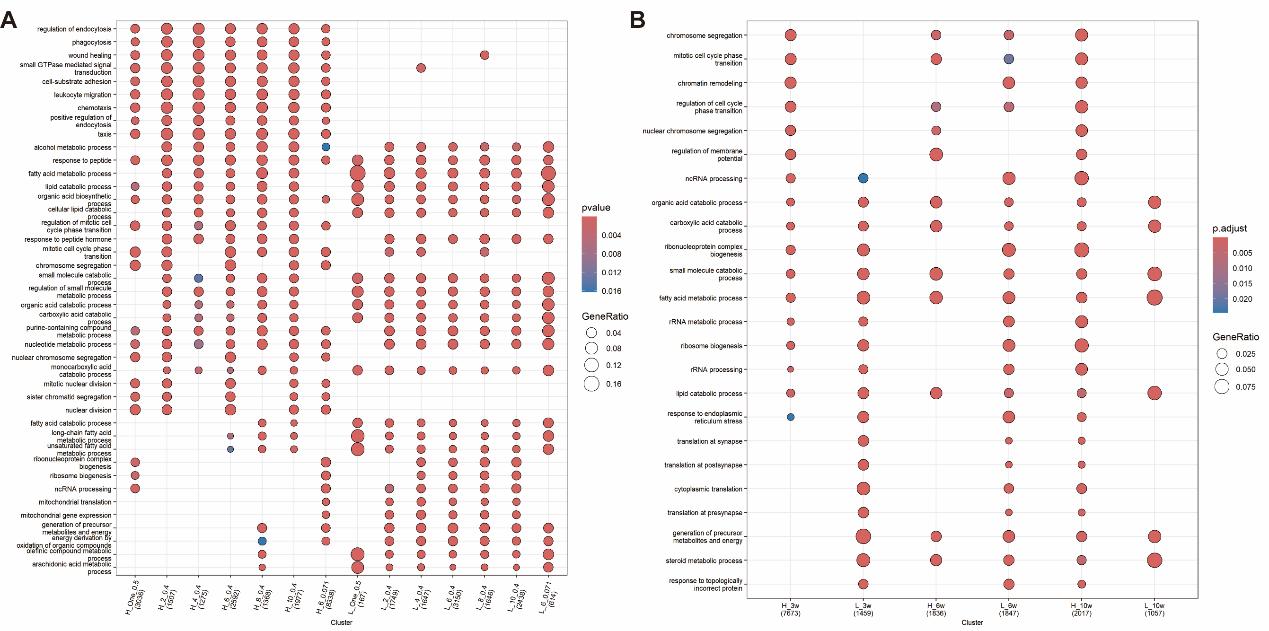


Supplementary Figure 2| GO enrichment analysis of differential genes in RNA-seq and scRNA-seq. A, GO enrichment bubble plot of differential genes in RNA-seq. B, GO enrichment bubble plot of differential genes in scRNA-seq.


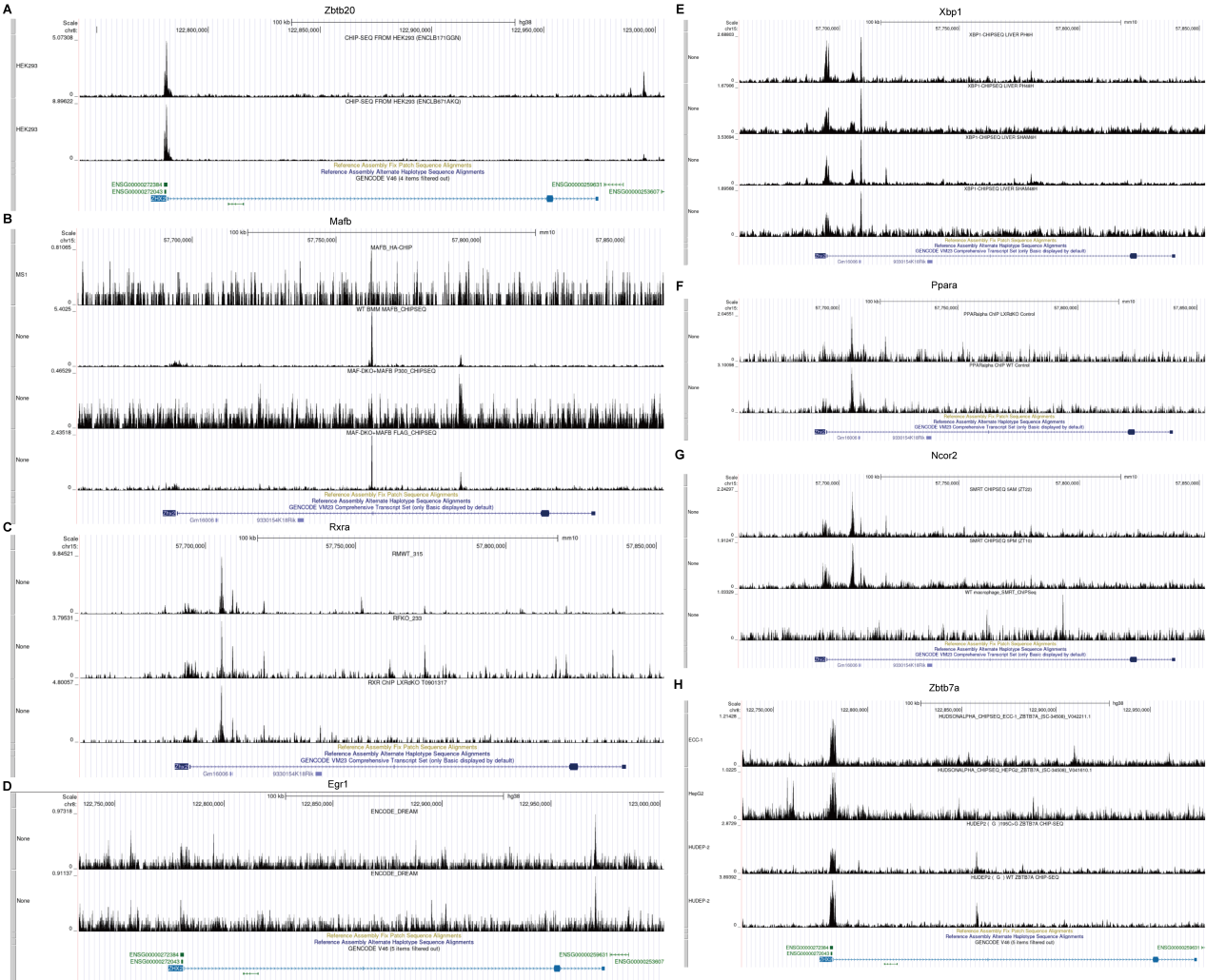


Supplementary Figure 3 | Chip-seq tracks of transcription factors. (A) Zbtb20 (human), (B) Mafb (mouse), (C) Rxra (mouse), (D) Egr1 (human), (E) Xbp1 (mouse), (F) Ppara (mouse), (G) Ncor2 (mouse) and (H) Zbtb7a (human).

Supplementary Table 1. Differential expression analysis of histone methylation genes in single cell data

| Group | Gene | avg_log2FC | pct.1 | pct.2 | p_val_adj |
| --- | --- | --- | --- | --- | --- |
| CCl4-3-0.1 | *Ash1l* | 0.887661 | 0.511 | 0.315 | 9.92E-105 |
|  | *Ash2l* | 0.981719 | 0.061 | 0.028 | 7.09E-13 |
|  | *Auts2* | 4.055308 | 0.142 | 0.009 | 3.24E-149 |
|  | *Baz2a* | 0.732638 | 0.267 | 0.169 | 1.34E-32 |
|  | *Dot1l* | 1.282283 | 0.176 | 0.077 | 6.37E-53 |
|  | *Eed* | 0.578211 | 0.246 | 0.151 | 2.56E-30 |
|  | *Ehmt1* | 0.879493 | 0.435 | 0.272 | 2.38E-76 |
|  | *Ehmt2* | 0.451583 | 0.142 | 0.099 | 1.24E-07 |
|  | *Ezh1* | 0.567369 | 0.084 | 0.052 | 5.63E-07 |
|  | *Ezh2* | 2.700454 | 0.151 | 0.03 | 2.37E-107 |
|  | *Gcgr* | -0.46351 | 0.567 | 0.688 | 3.30E-54 |
|  | *Kansl1* | 0.938365 | 0.662 | 0.449 | 4.15E-159 |
|  | *Kansl2* | 0.411987 | 0.231 | 0.17 | 9.01E-12 |
|  | *Kansl3* | 0.326453 | 0.22 | 0.172 | 1.65E-05 |
|  | *Kmt2a* | 0.62867 | 0.265 | 0.181 | 8.88E-24 |
|  | *Kmt2b* | 0.903832 | 0.106 | 0.054 | 1.26E-19 |
|  | *Kmt5b* | 0.811801 | 0.233 | 0.135 | 2.50E-37 |
|  | *Kmt5c* | 0.586342 | 0.119 | 0.078 | 7.77E-09 |
|  | *Mcrs1* | 0.985018 | 0.053 | 0.026 | 6.46E-09 |
|  | *Mecp2* | 0.892604 | 0.154 | 0.086 | 2.95E-24 |
|  | *Nelfa* | 0.794217 | 0.08 | 0.045 | 3.42E-10 |
|  | *Ogt* | 0.945001 | 0.471 | 0.286 | 2.33E-101 |
|  | *Phf20* | 1.477898 | 0.188 | 0.072 | 5.56E-70 |
|  | *Prdm6* | 3.236343 | 0.021 | 0.002 | 5.73E-16 |
|  | *Prdm9* | 1.18691 | 0.071 | 0.03 | 4.10E-19 |
|  | *Prmt5* | 0.733248 | 0.064 | 0.042 | 0.001637 |
|  | *Rbbp5* | 1.189034 | 0.11 | 0.047 | 1.65E-30 |
|  | *Setbp1* | 3.622352 | 0.068 | 0.006 | 4.69E-62 |
|  | *Setd1a* | 0.686523 | 0.154 | 0.097 | 6.34E-16 |
|  | *Setd1b* | 0.32626 | 0.109 | 0.081 | 0.026117 |
|  | *Setd2* | 0.97075 | 0.335 | 0.186 | 1.32E-72 |
|  | *Setd7* | 2.619896 | 0.084 | 0.014 | 9.27E-62 |
|  | *Setdb1* | 0.914542 | 0.27 | 0.144 | 1.47E-57 |
|  | *Setdb2* | 0.512674 | 0.235 | 0.201 | 0.042665 |
|  | *Smarca5* | 0.988605 | 0.121 | 0.06 | 1.66E-24 |
|  | *Smyd1* | 2.241131 | 0.065 | 0.014 | 4.11E-38 |
|  | *Smyd3* | 1.684958 | 0.245 | 0.085 | 5.03E-112 |
|  | *Smyd5* | 0.886273 | 0.027 | 0.013 | 0.003335 |
|  | *Suv39h1* | 1.896004 | 0.019 | 0.006 | 5.25E-06 |
|  | *Suv39h2* | 1.913351 | 0.012 | 0.003 | 0.006618 |
|  | *Suz12* | 1.36932 | 0.236 | 0.094 | 5.14E-87 |
| CCl4-6-0.1 | *Auts2* | 2.943552 | 0.046 | 0.009 | 1.79E-31 |
|  | *Dot1l* | -0.70856 | 0.079 | 0.139 | 2.06E-09 |
|  | *Ehmt1* | -0.24878 | 0.336 | 0.426 | 5.94E-08 |
|  | *Gcgr* | -0.45973 | 0.628 | 0.766 | 1.12E-40 |
|  | *Kansl1* | 0.362444 | 0.623 | 0.582 | 2.76E-07 |
|  | *Kmt2a* | -0.49015 | 0.18 | 0.265 | 3.78E-11 |
|  | *Kmt2b* | -0.67235 | 0.065 | 0.11 | 1.80E-05 |
|  | *Kmt5b* | -0.36919 | 0.149 | 0.204 | 0.000437 |
|  | *Ogt* | 0.489489 | 0.465 | 0.4 | 2.39E-09 |
|  | *Prmt5* | -0.78064 | 0.038 | 0.069 | 0.002539 |
|  | *Setbp1* | 2.196071 | 0.026 | 0.006 | 2.99E-12 |
|  | *Setd7* | 1.5475 | 0.06 | 0.024 | 1.55E-14 |
|  | *Setdb2* | 1.106069 | 0.335 | 0.214 | 7.43E-37 |
|  | *Wdr5* | -0.45469 | 0.094 | 0.148 | 1.50E-06 |
|  | *Xbp1* | -0.85513 | 0.208 | 0.389 | 6.55E-49 |
| CCl4-10-0.1 | *Auts2* | 1.888906 | 0.048 | 0.015 | 1.40E-08 |
|  | *Baz2a* | 0.466785 | 0.278 | 0.22 | 0.019215 |
|  | *Dot1l* | 0.650183 | 0.166 | 0.112 | 0.000184 |
|  | *Ehmt2* | 0.698287 | 0.203 | 0.134 | 1.87E-07 |
|  | *Kansl1* | -0.36779 | 0.54 | 0.626 | 5.57E-07 |
|  | *Kansl2* | 0.672274 | 0.304 | 0.221 | 2.60E-09 |
|  | *Kmt5c* | 1.281004 | 0.186 | 0.099 | 1.35E-16 |
|  | *Mcrs1* | 0.964518 | 0.066 | 0.033 | 0.000482 |
|  | *Ogt* | -0.48169 | 0.363 | 0.471 | 6.97E-11 |
|  | *Prmt5* | 1.358493 | 0.089 | 0.039 | 3.87E-10 |
|  | *Setd1a* | 0.993502 | 0.229 | 0.128 | 7.42E-19 |
|  | *Setd7* | 3.38777 | 0.079 | 0.01 | 3.79E-37 |
|  | *Setdb1* | 0.750605 | 0.323 | 0.223 | 1.28E-13 |
|  | *Setdb2* | -0.68735 | 0.193 | 0.279 | 6.33E-08 |
|  | *Smyd2* | 1.241029 | 0.116 | 0.056 | 1.79E-11 |
|  | *Smyd5* | 1.741687 | 0.05 | 0.015 | 3.80E-09 |
|  | *Wdr5* | 0.772059 | 0.178 | 0.117 | 3.95E-06 |
